# Lung Volume Reduction following Recurrent Pneumonia: An Unusual Finding in a COPD Patient

**DOI:** 10.1155/2017/7176816

**Published:** 2017-03-08

**Authors:** Yihenew Negatu, Philip T. Diaz

**Affiliations:** Division of Pulmonary, Critical Care and Sleep Medicine, The Ohio State University Wexner Medical Center, Columbus, OH, USA

## Abstract

Chronic Obstructive Pulmonary Disease (COPD) is a progressive disease. Frequent pneumonias and exacerbations are known to accelerate its progression. We present a case of severe emphysema whose lung function paradoxically improved following recurrent pneumonia, without lung volume reduction surgery (LVRS). A 54-year-old female with severe COPD presented for LVRS evaluation. She was not a candidate for the surgery because of the unsuitable anatomic distribution of her emphysema. The patient experienced recurrent pneumonia over the years but her lung function and oxygen requirement showed marked improvement. Follow-up imaging studies showed decreased lung volumes and focal fibrotic changes. We believe that the improvement in her lung function overtime is the reflection of lung volume reduction as a result of parenchymal remodeling due to repeated lung infection. These findings seen in our patient contribute important information for the continued effort in developing nonsurgical lung volume reduction techniques.

## 1. Introduction

Chronic Obstructive Pulmonary Disease (COPD) is a major cause of chronic morbidity and the fourth leading cause of death worldwide. It is characterized by persistent airflow limitation that is usually progressive. Exacerbations are characteristic of the course of COPD and contribute to the overall severity of the disease [1]. Nearly 70–80% of COPD exacerbations are triggered by viral and bacterial infections. Recurrent exacerbations are associated with accelerated decline in lung function [2].

We present an unusual case of a patient with severe emphysema whose forced expiratory volume in 1 second (FEV1), lung volumes, and blood gases improved markedly following recurrent exacerbations, characterized by repeated pneumonia.

## 2. Case Presentation

**Figure 1:**
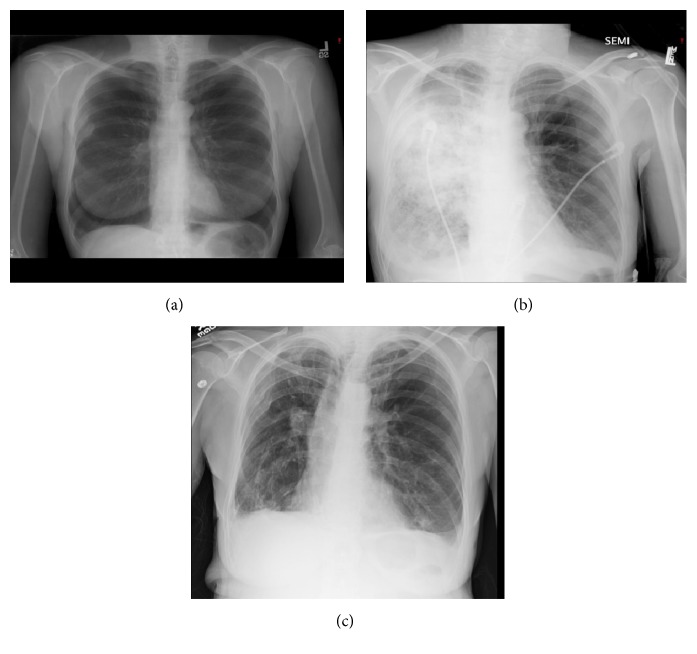
Baseline (a), during pseudomonas pneumonia (b), and follow-up (c) chest x-rays.

**Figure 2:**
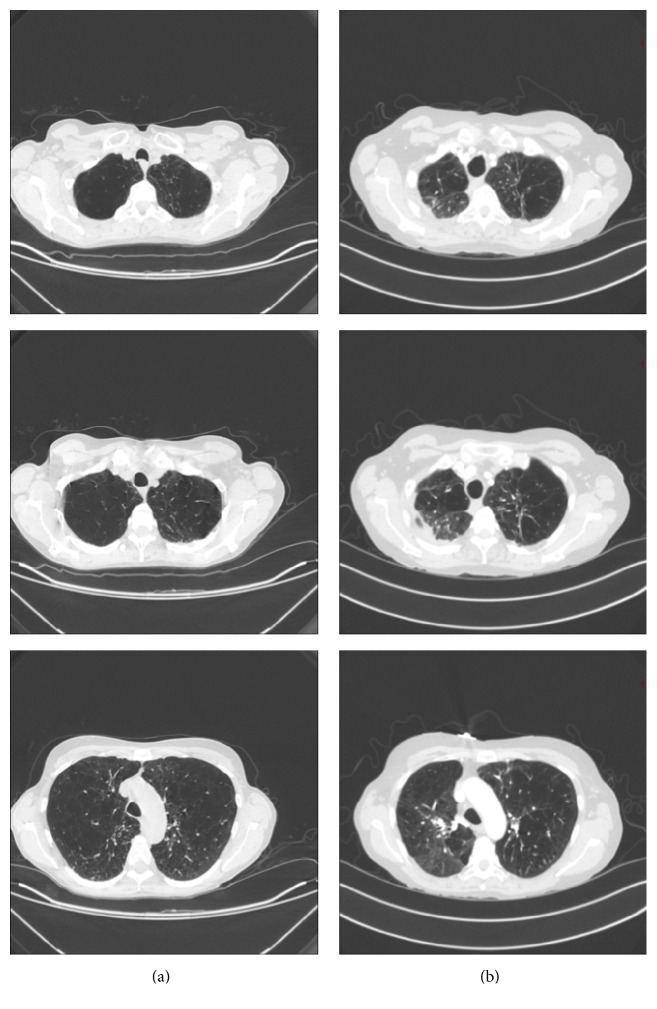
Baseline CT scan (a) which shows diffuse emphysema and a follow-up CT (b) which shows apical scarring predominantly on the right and right apical pleural thickening.

A 54-year-old female with sixty pack-year smoking history who quit smoking in 2006, severe airflow obstruction, and marked air-trapping was evaluated at our institution in April 2007 for LVRS but was deemed not a candidate because of a homogeneous distribution of her emphysema on chest imaging. Two months after evaluation she developed pseudomonas pneumonia with extensive involvement of her right lung. Bronchoscopy during this admission revealed diffuse erythema and purulent secretion with no evidence of endobronchial lesions or structural distortions. Her course was complicated by prolonged respiratory failure requiring tracheostomy and pneumothorax with prolonged air-leak. The patient was discharged after a two-month hospital stay and had gradual improvement in her respiratory status over the next three years, despite three additional hospital admissions for pneumonia over that time period. The right lung was predominantly involved in all of these episodes even though the left lung was not completely spared. By March 2010 she was able to be weaned off of oxygen. In December 2010 she had repeat pulmonary function studies performed which showed a dramatic improvement in her FEV1, lung volumes, and arterial blood gases (Table 1). Her imaging studies showed fibrotic changes, right apical pleural thickening, and decreased lung volume compared to her baseline studies which demonstrated marked hyperinflation and diaphragm flattening (Figures 1 and 2). Throughout her follow-up she was on maximal medical therapy which included long acting anticholinergic, long acting beta agonist, inhaled glucocorticoids, and short acting bronchodilator as needed.

## 3. Discussion

It is widely accepted that an accelerated rate of decline in FEV1 is a fundamental feature in the natural course of COPD [3, 4] and this decline is typically more pronounced among those who experience frequent exacerbations [5, 6].

Our patient, on the contrary, showed significant improvement in FEV1 and blood gases over time despite frequent exacerbations characterized by recurrent pneumonia. These findings in our patient are likely due to a reduction in lung volumes as a result of scarring and remodeling following recurrent infection. The scarring was clearly evidenced on serial imaging studies of her lungs. Lung volume reduction in general is thought to improve lung function primarily by better matching the size of the lungs to the thorax which in turn improves expiratory airflow and reduces dynamic and static hyperinflation. Reduced lung volumes also allow the inspiratory muscles to lower pleural pressure with greater mechanical efficiency [7]. This may result in improvement in ventilation and reduction in hypercarbia.

It is known that LVRS improves spirometry, lung volumes, and symptoms. The National Emphysema Treatment Trial showed that LVRS resulted in improvement of exercise capacity, health-related quality of life, and long-term survival in subset of patients with upper lobe predominant emphysema and a low exercise capacity. However, it is major surgery associated with significant morbidity and in some patients, surgical intervention is not even an option because of comorbidities [8]. Hence, there has been a continued effort to develop safe and effective nonsurgical techniques. In recent years, evidence has emerged suggesting that biologic lung volume reduction may be a feasible and safe technique. This technique involves bronchoscopic guided instillation of biological reagents to distal airway with the aim of inducing inflammation and eventually remodeling and shrinkage of damaged regions of lung [9, 10]. While studies done examining this technique are limited, some have shown promising results [11, 12]. Our case represents a “proof of concept” supporting the notion that biologic agents triggering similar structural changes could reduce lung volumes and improve functional status.

## 4. Conclusion

This case demonstrates dramatic improvement in lung function following recurrent pneumonia in a patient with severe emphysema. We believe that this example provides important insight for the continued search of effective nonsurgical LVR methods.

## Figures and Tables

**Table 1:** Lung function tests over time.

Parameters	April 2007	March 2009	December 2010
Forced vital capacity (FVC), L (% of predicted)	1.52 (53)	2.78 (97)	2.79 (97)
Forced expiratory volume in 1 second (FEV1), L (% of predicted)	0.50 (21)	1.03 (44)	1.16 (50)
FEV1/FVC	0.33	0.34	0.42
Total lung capacity (TLC), L (% of predicted)	6.69 (152)		5.51 (116)
Residual volume (RV), L (% of predicted)	5.47 (313)		2.81 (151)
PO_2_ (mmHg)	63		77
PCO_2_ (mmHg)	52		34

## Abbreviations

COPD: Chronic Obstructive Pulmonary Disease
FEV1: Forced expiratory volume in 1 second
LVRS: Lung volume reduction surgery.
